# Skeletal Muscle Mass Index Is Positively Associated With Bone Mineral Density in Hemodialysis Patients

**DOI:** 10.3389/fmed.2020.00187

**Published:** 2020-05-15

**Authors:** Kiyonori Ito, Susumu Ookawara, Yutaka Hibino, Sojiro Imai, Mariko Fueki, Yusaku Bandai, Masatoshi Yasuda, Tatsuya Kamimura, Hideo Kakuda, Satoshi Kiryu, Noriko Wada, Yuri Hamashima, Tadanao Kobayashi, Mitsutoshi Shindo, Hidenori Sanayama, Yasushi Ohnishi, Kaoru Tabei, Yoshiyuki Morishita

**Affiliations:** ^1^Division of Nephrology, Department of Integrated Medicine, Saitama Medical Center, Jichi Medical University, Saitama, Japan; ^2^Department of Orthopaedic Surgery, Minami-Uonuma City Hospital, Niigata, Japan; ^3^Department of Dialysis, Minami-Uonuma City Hospital, Niigata, Japan; ^4^Department of Radiology, Minami-Uonuma City Hospital, Niigata, Japan; ^5^Department of Population Health Science, Bristol Medical School, University of Bristol, Bristol, United Kingdom; ^6^Department of Internal Medicine, Yuzawa-Machi Health Medical Center, Niigata, Japan; ^7^Department of Neurology, Department of Integrated Medicine, Saitama Medical Center, Jichi Medical University, Saitama, Japan; ^8^Department of Internal Medicine, Minami-Uonuma City Hospital, Niigata, Japan

**Keywords:** bone mineral density, skeletal muscle mass index, hemodialysis, sarcopenia, osteoporosis, osteosarcopenia

## Abstract

**Background:** Patients with chronic kidney disease (CKD) are at risk for bone loss and sarcopenia because of associated mineral and bone disorders (MBD), malnutrition, and chronic inflammation. Both osteoporosis and sarcopenia are associated with a poor prognosis; however, few studies have evaluated the relationship between muscle mass and bone mineral density (BMD) in hemodialysis (HD) patients. The present study examined the association between skeletal muscle mass index (SMI) and BMD in the lumbar spine and femoral neck in HD patients.

**Methods:** Fifty HD patients (mean age, 69 ± 10 years; mean HD duration, 9.0 ± 8.8 years) in Minami-Uonuma City Hospital were evaluated. BMD was measured by dual-energy X-ray absorptiometry, and SMI was measured by bioelectrical impedance analysis (InBody^TM^) after HD. The factors affecting lumbar spine and femoral neck BMD were investigated, and multivariate analysis was performed.

**Results:** In simple linear regression analysis, the factors that significantly affected the lumbar spine BMD were sex, presence of hypertension, presence of diabetes mellitus, body mass index, triglyceride level, grip strength, and SMI; the factors that significantly affected the femoral neck BMD were sex, HD duration, serum creatinine level, tartrate-resistant acid phosphatase 5b level, undercarboxylated osteocalcin (ucOC) level, N-terminal propeptide of type I procollagen level, grip strength, and SMI. In multivariate analysis, SMI (standardized coefficient: 0.578) was the only independent factor that affected the lumbar spine BMD; the independent factors that affected the femoral neck BMD were SMI (standardized coefficient: 0.468), ucOC (standardized coefficient: −0.366) and sex (standardized coefficient: 0.231).

**Conclusion:** SMI was independently associated with the BMD in the lumbar spine and femoral neck in HD patients. The preservation of skeletal muscle mass could be important to prevent BMD decrease in HD patients, in addition to the management of CKD-MBD.

## Introduction

Low bone mineral density (BMD) is common in patients with end-stage renal disease and chronic kidney disease (CKD), and hemodialysis (HD) increases the risk for bone loss and fractures. Low BMD is associated with the occurrence of new fractures, reduced quality of life, and increased mortality (1, 2). Thus, it is important to manage mineral and bone disorders (MBD) associated with CKD (CKD-MBD) (3). Additionally, HD patients commonly have decreased skeletal muscle mass and decreased muscle strength, known as sarcopenia (4). Both osteoporosis and sarcopenia are reportedly associated with poor mortality (4, 5). However, few studies have evaluated the association between BMD and skeletal muscle mass. Furthermore, some reports have shown that muscle mass is positively associated with BMD (6, 7), while other studies have not found this positive association (8). Therefore, the relationship between BMD and muscle mass in HD patients remains unclear. The present study examined the association between skeletal muscle mass index (SMI) and BMD in the lumbar spine and femoral neck in HD patients.

## Materials and Methods

The present study included patients with end-stage renal disease treated with intermittent HD who were older than 20 years. Patients with implanted pacemakers were excluded, as the presence of pacemaker prevents body fluid composition measurement using bioelectrical impedance analysis (BIA).

Fifty HD patients were recruited (31 men, 19 women; mean age, 69 ± 10 years; mean HD duration, 9.0 ± 8.8 years). The patients' characteristics are summarized in Table 1. The causes of chronic renal failure were diabetes mellitus (17 patients), chronic glomerulonephritis (19 patients), nephrosclerosis (3 patients), and other (11 patients). Each patient underwent maintenance HD three times per week for 3–5 h in each session. Informed consent for study inclusion was obtained from each patient. The study was approved by the Institutional Review Board of Minami-Uonuma City Hospital (H29-12), Japan, and conformed to the provisions of the Declaration of Helsinki (revised in Tokyo in 2004).

**Table 1:** Patient characteristics.

**Characteristics**	***n* = 50**
Sex male, *n* (%)	31 (64)
Age (years)	69 ± 10
HD duration (year)	9.0 ± 8.8
**Primary disease**, ***n*** **(%)**	
Diabetes mellitus	17 (34)
Nephrosclerosis	3 (6)
Chronic glomerulonephritis	19 (38)
Others	11 (22)
**Past medical history**, ***n*** **(%)**	
Hypertension	44 (88)
Diabetes mellitus	20 (40)
Bone fracture	9 (18)
Ischemic heart disease	8 (16)
**Medication**, ***n*** **(%)**	
Vitamin D	34 (68)
*P* binder	42 (84)
Calcimimetics	11 (22)
Body mass index (kg/m^2^)	22.1 ± 4.6
Systolic BP (mmHg)	150 ± 23
Diastolic BP (mmHg)	78 ± 12
Heart rate (/min)	69 ± 12
**Laboratory findings**	
Albumin (g/dL)	3.5 ± 0.4
Serum sodium (mEq/L)	138 ± 3
Serum potassium (mEq/L)	4.9 ± 0.7
Serum calcium, mg/dL	8.4 ± 0.6
Serum phosphate (mg/dL)	5.1 ± 1.1
BUN (mg/dL)	60 ± 13
Cr (mg/dL)	10.1 ± 1.1
Hb (g/dL)	11.2 ± 0.9
Total cholesterol (mg/dL)	156 ± 41
Triglyceride (mg/dL)	109 ± 68
C-reactive protein (mg/dL)	0.5 ± 0.9
Intact PTH (pg/mL)	154 ± 109
ALP (U/L)	245 ± 101
BAP (μg/mL)	16 ± 13
TRACP-5b (mU/dL)	491 ± 240
ucOC (ng/mL)	31 ± 15
Total P1NP (ng/mL)	257 ± 154
**Physical status and BMD**	
Grip strength (kg)	23 ± 8
Skeletal muscle mass index (kg/m^2^)	6.1 ± 1.2
Lumbar spine bone density (g/cm^2^)	0.91 ± 0.21
Proxymal femur bone density (g/cm^2^)	0.56 ± 0.12

*HD, hemodialysis; BP, blood pressure; BUN, blood urea nitrogen; Cr, creatinine; Hb, hemoglobin; PTH, parathyroid hormone; ALP, alkaline phosphatase; BAP, bone specific alkaline phosphatase; TRACP-5b, tartrate-resistant acid phosphatase 5b; ucOC, undercarboxylated osteocalcin; P1NP, N-terminal propeptide of type I procollagen; BMD, bone mineral density*.

### Data Collection

Baseline patient characteristics and other relevant data, including the primary disease of CKD, were collected from the medical records. Blood pressure and pulse rate were measured with patients in the supine position before the HD session at which each SMI measurement was taken. Body mass index (BMI) was calculated using the dry weight (DW). Blood samples were obtained from the arteriovenous fistula before HD.

### Measurement of SMI, BMD, and Grip Strength

SMI was calculated using body composition measurements obtained with multi-frequency BIA (InBody S10; InBody Japan, Tokyo, Japan). A skeletal muscle mass evaluation was performed after the last HD session; that is, patients were measured under DW so that the weight was not affected by the amount of body fluid. Then, the SMI was calculated by normalizing the skeletal muscle mass for height (kg/m^2^).

Dual energy X-ray absorptiometry (Horizon A, HOLOGIC Japan, Tokyo, Japan) was used to evaluate the BMD in the lumbar spine (L2–L4) and femoral neck. To minimize variations in BMD measurements, a single radiologic technologist performed scanning and BMD data calculations for all HD patients.

Grip strength was measured by a grip strength meter (Smedley's Hand Dynamo Meter, Matsumiya Ika Seiki, Tokyo, Japan). Grip strength was measured twice for the hand without a dialysis shunt before the HD session. The mean of the two measurements was used as the grip strength for each patient.

### Statistics

Data are expressed as mean ± standard deviation. The paired Student's *t*-test was used to compare two parameters. Inter-group correlations were evaluated by Pearson's correlation coefficient and linear regression analysis. Multivariate linear regression analysis was performed using the parameters with statistically significant correlations in univariate analyses (*p* < 0.05) to determine the independent factors that influenced the lumbar spine BMD and femoral neck BMD. All analyses were performed using SPSS Statistics version 19.0 (IBM, Armonk, NY, USA). Differences with a *p* < 0.05 were considered statistically significant.

## Results

The clinical parameters of the study participants are shown in Table 1. The lumbar spine BMD in all included HD patients was 0.91 ± 0.21 g/cm^2^. The lumbar spine BMD was significantly greater in men (0.96 ± 0.21 g/cm^2^) than in women (0.80 ± 0.20 g/cm^2^, *p* < 0.01). The female neck BMD in all included HD patients was 0.56 ± 0.12 g/cm^2^. The femoral neck BMD was significantly greater in men (0.60 ± 0.12 g/cm^2^) than in women (0.49 ± 0.07 g/ cm^2^, *p* < 0.01). In addition, the lumbar spine BMD was significantly greater than the femoral neck BMD (*p* < 0.001). The SMI in all patients was 6.10 ± 1.20 kg/m^2^, and the grip strength was 23 ± 8 kg.

**Figure 1:**
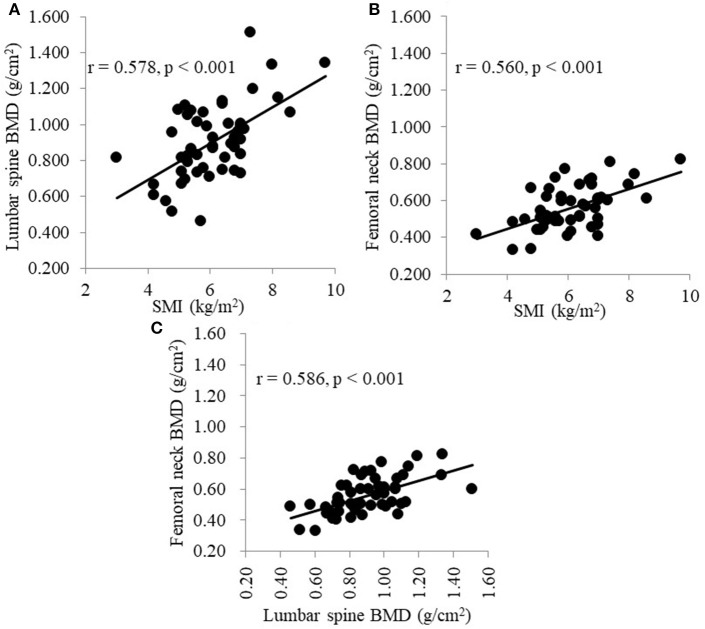
**(A)** Correlation between SMI and the BMD of the lumbar spine. **(B)** Correlation between SMI and the BMD of the femoral neck. **(C)** Correlation between the BMD of the lumbar spine and the BMD of the femoral neck. SMI, skeletal muscle mass index; BMD, bone mineral density.

Table 2 shows the correlations between each BMD and the clinical parameters. Simple linear regression analysis showed that the factors that significantly affected the lumbar spine BMD were sex, presence of hypertension, presence of diabetes mellitus, BMI, triglyceride level, grip strength, and SMI; the factors that significantly affected the femoral neck BMD were sex, HD duration, serum creatinine level, tartrate-resistant acid phosphatase 5b level, undercarboxylated osteocalcin (ucOC) level, total N-terminal propeptide of type I procollagen level, grip strength, and SMI. Figure 1 shows that SMI was positively correlated with both the lumbar spine BMD (*r* = 0.578, *p* < 0.001) and the femoral neck BMD (*r* = 0.560, *p* < 0.001). Additionally, the lumbar spine BMD was positively correlated with femoral neck BMD (*r* = 0.586, *p* < 0.001; Figure 1C).

**Table 2:** Correlations between clinical parameters and respective BMD.

	**Lumbar spine**		**Femoral neck**	
	***r***	***p*-value**	***r***	***p*-value**
Sex male, *n* (%)	0.356	0.011	0.416	0.003
Age (years)	−0.266	0.062	−0.275	0.053
HD duration (year)	−0.231	0.106	−0.311	0.027
**Past medical history**, ***n*** **(%)**				
Hypertension	0.283	0.046	0.178	0.216
Diabetes mellitus	0.330	0.019	0.178	0.216
Bone fracture	−0.227	0.112	−0.258	0.078
Ischemic heart disease	−0.147	0.308	−0.005	0.970
Body mass index (kg/m^2^)	0.435	0.002	0.270	0.058
Systolic BP (mmHg)	0.051	0.725	−0.078	0.591
Diastolic BP (mmHg)	−0.178	0.217	−0.195	0.175
Heart rate (/min)	−0.105	0.486	−0.172	0.232
Albumin (g/dL)	0.037	0.800	0.046	0.750
Serum sodium (mEq/L)	−0.065	0.652	−0.261	0.167
Serum potassium (mEq/L)	−0.199	0.166	0.018	0.899
Serum calcium (mg/dL)	0.111	0.443	0.061	0.675
Serum phosphate (mg/dL)	0.054	0.707	0.107	0.461
BUN (mg/dL)	−0.052	0.718	0.072	0.620
Cr (mg/dL)	0.227	0.113	0.301	0.034
Hb (g/dL)	−0.011	0.939	−0.036	0.802
Total cholesterol (mg/dL)	0.096	0.508	0.111	0.443
Triglyceride (mg/dL)	0.298	0.035	0.278	0.051
C-reactive protein (mg/dL)	0.036	0.801	−0.005	0.972
intact PTH (pg/mL)	−0.017	0.905	−0.182	0.205
ALP (U/L)	−0.075	0.606	−0.209	0.146
BAP (μg/mL)	−0.210	0.143	−0.233	0.103
TRACP-5b (mU/dL)	−0.199	0.180	−0.419	0.002
ucOC (ng/mL)	−0.175	0.223	−0.382	0.006
total P1NP (ng/mL)	−0.145	0.314	−0.380	0.006
Grip strength (kg)	0.471	0.001	0.507	<0.001
Skeletal muscle mass index (kg/m^2^)	0.578	<0.001	0.560	<0.001

*HD, hemodialysis; BP, blood pressure; BUN, blood urea nitrogen; Cr, creatinine; Hb, hemoglobin; PTH, parathyroid hormone; ALP, alkaline phosphatase; BAP, bone specific alkaline phosphatase; TRACP-5b, tartrate-resistant acid phosphatase 5b; ucOC, undercarboxylated osteocalcin; P1NP, N-terminal propeptide of type I procollagen; BMD, bone mineral density*.

In multivariate analysis, the only factor that significantly affected the lumbar spine BMD was the SMI (standardized coefficient: 0.578), while the femoral neck BMD was significantly affected by the SMI (standardized coefficient: 0.468), ucOC level (standardized coefficient: −0.366), and sex (standardized coefficient: 0.231; Table 3).

**Table 3:** Multivariate regression analysis: Independent factors of BMD in the lumbar spine and femoral neck.

**BMD**	**Variables**	**Coefficient**	**Standardized coefficient**	***p*-value**
Lumbar spine	SMI	0.101	0.578	<0.001
Femoral neck	SMI	0.046	0.468	<0.001
	ucOC	−0.003	−0.366	0.001
	Sex (male:1)	0.055	0.231	0.046

*BMD, bone mineral density; SMI, skeletal muscle mass index; ucOC, undercarboxylated osteocalcin*.

## Discussion

The present study investigated the association between SMI and the BMD of the lumbar spine and the femoral neck. It is well-known that the risk of bone fracture is increased in dialysis patients (1, 9), and malnutrition and sarcopenia are associated with poor prognosis (10, 11). However, reports regarding the association between osteoporosis and sarcopenia in HD patients are limited (6, 7, 12). Similarly to these previous reports, our results demonstrated a positive correlation between SMI and BMD. Muscle, especially skeletal muscle, supports the bones and positively supports physical activity (13). Therefore, it is important to understand osteosarcopenia in HD patients with both CKD-MBD and malnutrition.

Osteosarcopenia is a new concept that includes both osteoporosis and sarcopenia, and leads to an increased risk of frailty (14). The incidence of osteosarcopenia in general population is reportedly 5.8% (15) and this incidence is likely to be even higher in HD patients. The prevalence of osteosarcopenia in patients with CKD who have undergone kidney transplantation is reportedly 17.2%, and these patients have a slower walking speed than patients without osteosarcopenia (16). In contrast, exercise contributes to BMD preservation (17), and exercise training improves grip strength and walking speed in dialysis patients (18). Therefore, the presence of osteosarcopenia, which decreases the ability to exercise, might be associated with negative cycles involving bone, skeletal muscle, and physical activity. In the clinical setting, HD patients must be properly managed, including the management of CKD-MBD to prevent both osteoporosis and sarcopenia.

In the present study, there were no correlation found between BMD and the levels of serum calcium, serum phosphate, and intact parathyroid hormone. Furthermore, BMD was not associated with bone turnover or osteogenesis markers. However, various therapeutic agents were used to appropriately manage the CKD-MBD status of patients undergoing HD and the CKD-MBD related data was well controlled. This might be the reason that these makers were not found to be significant independent factors associated with BMD in the present study. In contrast, previous studies have found that some bone turnover or osteogenesis markers were independently associated with BMD (6, 7). These markers were also associated with the femoral neck BMD in simple liner regression performed in the present study. However, although bone metabolism-related makers were not associated with BMD, SMI was independently associated with BMD. A previous study reported that the femoral neck BMD was affected by body weight and serum creatinine level, which could reflect muscle mass (19). Therefore, the maintenance of skeletal muscle might be important in preventing BMD. Further prospective studies are required to clarify the association between CKD-MBD or bone metabolism and BMD.

In the present study, the femoral neck BMD was significantly affected by the ucOC level. Osteocalcin is one of the factors related to bone formation, and is induced by vitamin K. In patients with vitamin K deficiency, osteocalcin is mainly present in the circulation as ucOC, which is reportedly inversely correlated with vitamin K (20). Furthermore, an increased ucOC level is a risk factor for hip fracture (21), and vitamin K levels are also associated with bone fracture in HD patients (22). Therefore, improving vitamin K deficiency could be important for the transformation of osteocalcin. However, HD patients should pay attention to their diet, even if they are vitamin K deficient, as foods such as vegetables, tea, and seaweed that contain vitamin K have high levels of potassium. Although vitamin K and ucOC are not routinely measured in dialysis clinics, these might be useful measurements for maintaining BMD or preventing bone fracture.

Although grip strength had a strong correlation with BMD in simple linear regression in the present study, it was not found to be an independent factor affecting the BMD of the lumbar spine or femoral neck because grip strength is reportedly a good predictor of overall muscle strength and a useful marker of frailty (23). Furthermore, the distal radius BMD is reportedly one of the determinants of handgrip strength (7). If the present study has evaluated the muscle strength in the lumbar spine or femur, muscle strength might have been found to be an independent factor affecting the BMD. Therefore, the association between muscle strength and BMD should be investigated in future studies.

The present study has some limitations. First, the sample size was relatively small. Second, this study was a cross-sectional study, and therefore, causal relationships cannot be confirmed. Third, as HD patients are at increased risk of bone fracture or mortality due to the influences of CKD-MBD, various medications used to treat CKD-MBD were prescribed throughout HD therapy. Therefore, a prospective longitudinal study is needed to investigate the associations between BMD and changes in CKD-MBD factors.

In conclusion, SMI was independently associated with the BMD of lumbar spine and femoral neck. Therefore, to prevent BMD decrease in HD patients, it may be important to preserve skeletal muscle mass in addition to performing CKD-MBD management.

## Data Availability Statement

The datasets generated for this study are available on request to the corresponding author.

## Ethics Statement

The studies involving human participants were reviewed and approved by the Institutional Review Board of Minami-Uonuma City Hospital (H29-12). The patients/participants provided their written informed consent to participate in this study.

## Author Contributions

KI conceived, designed, and performed the study, analyzed the data, and contributed to the writing of the manuscript. SO designed the study, analyzed the data, and contributed to the writing of the manuscript. YHi designed and performed the study, and supervised the writing of the manuscript. SI, MF, YB, MY, TKa, HK, SK, NW, YHa, TKo, and MS performed the study. HS, YO, KT, and YM supervised the writing of the manuscript. All authors reviewed the manuscript.

## Conflict of Interest

The authors declare that the research was conducted in the absence of any commercial or financial relationships that could be construed as a potential conflict of interest.
